# Rabbit manure compost as a peat substitute for compound growing media: Proportioning optimization according to physiochemical characteristics and seedling effects

**DOI:** 10.3389/fpls.2022.1008089

**Published:** 2022-10-27

**Authors:** Rangling Li, Hongyun Hao, Chengcai Yang, Liangju Wang, Hongying Wang

**Affiliations:** College of Engineering, China Agricultural University, Beijing, China

**Keywords:** waste management, sustainability, growing media, seedling quality, greenhouse gas emission, *Cucumis sativus*, *Calendula officinalis*

## Abstract

A large amount of rabbit manure is produced with the development of the rabbit industry, which will cause environmental pollution without proper treatment. Rabbit manure compost may be suitable for seedling cultivation, considering its low moisture, low heavy metal, high lignocellulose, and good fertilizer effect. In this study, a pre-proportioning test of growing media was conducted to optimize the ratio of perlite and vermiculite with peat/rabbit manure compost according to their physicochemical properties. Then, based on the results of the first proportioning optimization, the mixing ratio of rabbit manure compost and peat was further optimized using a bioassay. In this bioassay, salt-tolerant calendula (*Calendula officinalis* L.) and salt-intolerant cucumber (*Cucumis sativus* L.) were selected as test plants. The seedling effects (e.g., seedling emergence percentage, plant growth parameters, plant biomass, and nutrient effects) were evaluated. It was shown in the results that the rabbit manure compound growing media could be used for the seedlings, and suitable seedling performance was obtained with the increase of the total porosity (5.0%–61.2%), organic matter content (8.3%–39.9%), and nutrient elements from the rabbit manure compost. From the perspective of seedling emergence, there was no significant difference between rabbit manure compound media and peat treatment, in which the highest emergence percentages were >90%. At the same time, the nutrient performance of plant aboveground was significantly increased in rabbit manure compound growing media compared to peat treatment. In particular, the contents of P and Mg were increased by 31%–141.4% and 80.4%–107.8% for calendula and by 82.6%–117.4% and 35.1%–67.6% for cucumber, respectively. It was indicated in the two-step optimization that the rabbit manure compost proportion of 30%–50% (that is, 60%–100% instead of peat) was more suitable. Additionally, the greenhouse gas emission could be reduced by using rabbit manure compost replacing peat, and the greenhouse gas emission reduction potential would be 3.65 × 10^5^–4.06 × 10^8^ kg CO_2_-equivalent/year in China, which has important ecological significance.

## 1 Introduction

In recent years, the competitiveness of the rabbit industry has become strong, especially in Hungary, France, and Spain, since rabbits are the second largest farmed species in Europe. Similarly, China and Argentina also have large rabbit breeding industries (Dinuccio et al., 2019; Li and Wu, 2021). Subsequently, a large quantity of rabbit manure is produced, which may cause environmental pollution if not treated effectively. Therefore, increased attention has been paid to the resource treatment and disposal of rabbit manure (Islas-Valdez et al., 2017).

The National Livestock Station of China recommends that livestock and poultry waste should be composted to manufacture growing media trays or growing media as one of the effective ways to recycle biomass (Meng et al., 2019; Feng et al., 2020; Xu et al., 2021). Composted rabbit manure is considered suitable for use as solid growing media because of its low moisture (means low costs on collection and transport), low heavy metal (means high plant safety), high lignocellulose (means good porosity), and high nutrition (means good fertilizer effect) (Pereira et al., 2020; Ikrarwati et al., 2021; Li et al., 2022). Mahmoud et al. (2014) explored the use of rabbit manure in organic tomato (*Solanum lycopersicum* L.) transplants and observed interesting fertilizer effects. Rabbit manure as a growing media also impacts lettuce (*Lactuca sativa* var. *ramosa* Hort.) seedlings production (Pereira et al., 2020). Cabanillas et al. (2013) found that vermicompost from rabbit manure could also be an effective alternative to urea in basil (*Ocimum basilicum* L.) production. The feasibility of rabbit manure as plant-growing media was verified preliminarily, especially its partial peat replacement. At the same time, peat, a commonly used solid-growing media material, is nonrenewable and has essential ecological features (e.g., carbon sequestration) (Alshehri et al., 2020; Xu et al., 2021). Therefore, the Chinese government banned peatland mining (Joosten et al., 2012). However, the Chinese demand for growing media materials is vast and persistent as a horticultural powerhouse. If peat can be replaced with rabbit manure, it will effectively deal with the rabbit manure issue and reduce greenhouse gas emissions (Farina et al., 2018; Meng et al., 2022).

However, using a single component as the growing media usually has certain limitations in physical and chemical properties. Preparing compound growing media using various components is necessary to obtain better properties (Di Lonardo et al., 2021). Likewise, the properties of rabbit manure compost need to be adjusted by other materials due to its deficiencies, such as high salinity (Pereira et al., 2020). Perlite and vermiculite are the most commonly used growing media components, especially in ventilation regulation and water retention performance (Vinci and Rapa, 2019; Meng et al., 2022). Zhang et al. (2012) prepared growing media by mixing peat and perlite at 1:1 (v/v). Çelebi (2019) used growth media designed from 71% perlite and 29% peat (v/v). Additionally, there are studies in which the ratio of peat:perlite:vermiculite was set to 2:1:1 or 3:1:1 (Li et al., 2022). Still, the mixing proportion is critical because the balance of comprehensive agronomic effects, economy, environmental protection, etc., should be considered (Chang et al., 2021). For practical application, therefore, it was necessary to optimize the proportion of rabbit manure compound growing media according to the characteristics of rabbit manure and its impact on plant growth. In addition, the high electrical conductivity (EC) value of rabbit manure may cause salt damage to plants. However, the effect of rabbit manure compost instead of peat on the seedling of salt-tolerant crops and salt-intolerant crops, along with their differences, has not been reported. Moreover, little information is available regarding the carbon reduction potential of rabbit manure compost as a substitute for peat.

In this study, the best mixing range of rabbit manure compost was obtained by two-step optimization: (1) A pre-proportioning growing media test was conducted to optimize the ratio of perlite and vermiculite with peat/rabbit manure compost. This is because the physiochemical characteristics, including bulk density, porosity, pH, and EC values of growing media, depend heavily on perlite and vermiculite (Vinci and Rapa, 2019; Chang et al., 2021). (2) Based on the results of the first proportioning optimization, the mixing ratio of rabbit manure compost and peat was further optimized in a bioassay. Salt-tolerant calendula (*Calendula officinalis* L.) and salt-intolerant cucumber (*Cucumis sativus* L.) were chosen for the bioassay. This is because calendula has significant medicinal and ornamental values. Additionally, it is adapted to the high pH and EC values of growing media (Gong et al., 2018). On the other hand, cucumbers are one of the most critical vegetable species worldwide and have broad market prospects (Łaźny et al., 2021). The seedling effects (e.g., seedling emergence percentage, plant growth parameters, plant biomass, and nutrient effects) of different proportions of calendula and cucumber were evaluated. (3) The correlation between the physiochemical characteristics of organic media and seedling performance was analyzed. Additionally, the potential greenhouse gas emission reduction of rabbit manure compost replacing peat in China was calculated and discussed. Furthermore, it provides some theoretical reference and support for promoting biomass as growing media.

## 2 Materials and methods

### 2.1 Raw materials

Calendula and cucumber seeds were purchased from Beilin Technology Co., Ltd. and the Institute of Vegetables and Flowers, Chinese Academy of Agricultural Sciences. The raw growing media materials included composted rabbit manure, peat, perlite, and vermiculite. Fresh rabbit manure was collected from a large-scale breeding farm in Jiyuan, Henan Province, China, in November 2020 and was composted for 28 days. After that, the compost was allowed to mature for 30 days. Finally, the germination index of the composed rabbit manure was >70%, meaning it was not toxic to plants (NY 525-2021). The heavy metal contents of Cr, As, Cd, Hg, and Pb in rabbit manure compost were 7.06, 4.90, 0.37, 0.04, and 12.62 mg/kg, respectively. These numbers were far lower than the limit of growing media for flowering trees and shrubs (LY/T 2700-2016). Peat (PINDSTRUP, Denmark), perlite (3–5 mm), and vermiculite (2–4 mm) were purchased from a local market. All materials were naturally air-dried and sieved (<5 mm). Their physical and chemical properties are shown in **Table 1**.

**Table 1:** Physical and chemical properties of tested growing media materials.

Materials	Moisture (%)	BD (g/cm^3^)	TP (%)	AP (%)	WHP (%)	pH	EC (mS/cm)
Peat	37.5	0.18	51.1	14.2	32.7	5.8	0.3
Vermiculite	2.4	0.24	71.8	11.4	60.1	7.3	0.1
Perlite	0.2	0.05	44.5	22.9	22.0	6.6	0.0
Rabbit manure compost	20.3	0.22	78.3	8.5	69.8	8.7	3.7

The properties include moisture, bulk density (BD), total porosity (TP), aeration porosity (AP), water holding porosity (WHP), pH, and electrical conductivity (EC).

### 2.2 Growing media pre-proportioning

Eight kinds of pre-proportioning growing media were manufactured, as shown in **Table 2**, to preliminarily optimize the growing media and explore the suitable mixing ratio of rabbit manure compost. In the growing media industry, it is more common to describe the growing media composition by volume rather than weight (Xiong et al., 2017; Meng et al., 2022). Therefore, we chose to describe it by volume. Among these, PT1–PT4 were peat treatments in which perlite, vermiculite, and peat were mixed with a ratio of 50:0:50, 33.3:33.3:33.3, 25:25:50, and 20:20:60, respectively; RMT1–RMT4 were rabbit manure treatments in which perlite, vermiculite, and rabbit manure compost were mixed with a ratio of 50:0:50, 33.3:33.3:33.3, 25:25:50, and 20:20:60, respectively.

**Table 2:** Rabbit manure growing media pre-proportioning.

	Perlite	Vermiculite	Peat	Rabbit manure compost
Peat treatment				
PT1	50%	0	50%	/
PT2	33.3%	33.3%	33.3%	
PT3	25%	25%	50%	
PT4	20%	20%	60%	
Rabbit manure treatment				
RMT1	50%	0	/	50%
RMT2	33.3%	33.3%		33.3%
RMT3	25%	25%		50%
RMT4	20%	20%		60%

PT1–PT4 were peat treatments in which perlite, vermiculite, and peat were mixed with a ratio of 50:0:50, 33.3:33.3:33.3, 25:25:50, and 20:20:60, respectively; RMT1–RMT4 were rabbit manure treatments in which perlite, vermiculite, and rabbit manure compost were mixed with a ratio of 50:0:50, 33.3:33.3:33.3, 25:25:50, and 20:20:60, respectively. Values are the volume ratio of growing media materials.

The pre-proportioning of growing media was optimized according to physical and chemical properties, including bulk density (BD), total porosity (TP), aeration porosity (AP), water holding porosity (WHP), pH value, and EC value. Since a single parameter was challenging to evaluate the merits of different growing media, the membership function value method was used to determine the optimal growing media formula (Meng et al., 2019). The comprehensive evaluation index (CEI) was calculated using the following equation (1):


(1) $$\;CEI_{i}=\frac{\sum_{j=1}^{n}Z_{i,j}}{n}$$

where *n* is the number of parameters; *Z_i, j_* is the membership function value of the *j*th parameter in the *i*th treatment. *Z_i, j_* is calculated by following equation (2) or equation (3) when the parameter was positively or negatively correlated with the quality of growing media.


(2) $$\;Z_{i,j}=\frac{X_{i,\;\;j}-X_{min,j}}{X_{max,\;j}-X_{min,j}}$$

(3) $$\;\;\;\;\;Z_{i,j}=1-\frac{X_{i,j}-X_{min,j}}{X_{max,j}-X_{min,j}}$$

where *X_i, j_* is the specific parameter value of the *j*th parameter in the *i*th growing media; *X_max, j_* and *X_min, j_* are the maximum and minimum values of the *j*th parameter in all growing media.

### 2.3 Growing media proportioning

According to the optimization results of the pre-proportioning test, the proportioning of 50% peat/rabbit manure compost + 25% perlite + 25% vermiculite (v/v/v) was selected. The mixing ratio of peat and rabbit manure compost was further optimized in a bioassay after the appropriate proportion of perlite and vermiculite was determined to explore the optimal effect of replacing peat with rabbit manure compost. First, six kinds of growing media were manufactured, as shown in **Table 3**. The ratios of rabbit manure compost were 0% (in CK), 10% (in T10), 20% (in T20), 30% (in T30), 40% (in T40), and 50% (in T50). Correspondingly, the ratio of peat decreased from 50% (in CK) to 0% (in T50). The basic physicochemical properties (e.g., BD, TP, AP, WHP, pH, and EC) and nutrient elements [total nitrogen (N), phosphorus (P), potassium (K), calcium (Ca), and magnesium (Mg)] of the growing media were determined. The seedling effects of different growing media were analyzed by plant bioassay.

**Table 3:** Manufacture of different treatments.

	Rabbit manure compost	Peat	Perlite	Vermiculite
CK	0%	50%	25%	25%
T10	10%	40%	25%	25%
T20	20%	30%	25%	25%
T30	30%	20%	25%	25%
T40	40%	10%	25%	25%
T50	50%	0%	25%	25%

CK was treatment in which rabbit manure compost, peat, perlite, and vermiculite were mixed with a ratio of 0:50:25:25; T10–T50 were treatments in which rabbit manure compost, peat, perlite, and vermiculite were mixed with a ratio of 10:40:25:25, 20:30:25:25, 30:20:25:25, 40:10:25:25, and 50:0:25:25. Values are the volume ratio of growing media materials.

BD, TP, AP, and WHP were determined using the ring knife method, as in Gong et al. (2018). The moisture content and organic matter (OM) of growing media were determined by the gravimetric method. The former was dried at 105°C for 24 h, and the latter was calcined at 600°C for 4 h. P, K, Ca, and Mg contents were measured using an inductively coupled plasma spectrometer (Agilent ICPOES730, USA). The content of N was determined by an Elemental Analyzer (Elementar Vario EL cube, German). The pH and EC values of the samples were analyzed at a ratio of manure to distilled water of 1:10 using a pH meter (Sartorius PB-10, Sartorius AG, Göttingen, Germany) and a portable conductivity meter (Leici DDB-303A, Shanghai Oustor Industrial Corp., Shanghai, China).

### 2.4 Plant bioassay

The seedling cultivation test of calendula and cucumber was conducted in light incubators (HYM-400-G, Shanghai Huyueming Scientific Instrument Co., Ltd., China). Calendula and cucumber seeds (after germination treatment) were sown in 128-cell and 72-cell polystyrene trays (1 seed/cell), according to the production demand of vegetable and flower seedlings. Six kinds of growing media were filled into the polystyrene tray in order, and each growing media was repeated four to five times. The seeded polystyrene tray was randomly placed in a light incubator. During the seedling cultivation, the temperature was set as 25°C during the day and 18°C at night. After the cotyledons were arched, the light was set at 15,000 lx of light for 12 h/day. Calendula and cucumber seedlings were watered every 2 days. The cultivation test was completed when the seedlings achieved transplanted levels on the 35th day for calendula and the 31st day for cucumber. No additional nutrient solution was used in this test.

Seedling cultivation was observed daily, and the calendula and cucumber seedling emergence percentages of different growing media were measured on the 5th, 10th, and 15th days. The growth characteristics (e.g., stem diameter, stem length, root length, and total chlorophyll content), biomass (seedlings fresh and dry weights), and the macronutrient (e.g., total N, P, K, Ca, and Mg) of the aboveground part were measured in the middle and late stages of seedling cultivation. The middle stage was on the 25th day for calendula and the 15th day for cucumber. The late stage was on the 35th day for calendula and the 31st day for cucumber. Among them, the stem diameter was measured at 1 cm from the media surface. Ten seedlings (for calendula) and eight seedlings (for cucumber) were randomly selected from each treatment and cleaned with deionized water. Then, the length and fresh weight of the aboveground and the belowground parts were measured. After that, the plant samples were killed green at 105°C for 15 min. Then, the samples were dried at 80°C until constant weight to obtain the dry weight of the aboveground and belowground parts. Additionally, samples were taken at the same position as plant leaves to extract and determine chlorophyll, according to the method reported by Chang et al. (2019). All parameters were repeated at least three times.

Finally, some critical plant parameters, which were significantly affected by rabbit manure, were selected to comprehensively evaluate the seedling effect of rabbit manure compound growing media with CEI. The calculation method for CEI was the same as that in *Growing media pre-proportioning*.

### 2.5 Statistical analysis

Spearman’s correlation analysis was used to explore the relationship between growing media properties and seedling effects. One-way ANOVA and Duncan’s test were used for variance analysis by SPSS 17.0. A *p*-value < 0.05 was considered a significant difference. Figures were produced using Origin 2021.

## 3 Results

### 3.1 Growing media optimization of pre-proportioning

The characteristics of the pre-proportioning growing media are shown in **Table 4**. The physical and chemical properties were significantly affected by the mixing ratio. For example, the highest AP was observed in PT1 and RMT1, where perlite was mixed with peat or rabbit manure compost at 1:1. A high AP means an adequate ventilatory ability. This would be beneficial to the root growth of seedlings. Nonetheless, from the perspective of avoiding salt stress, low pH and EC are needed. The lowest pH and EC values were found in PT3 and RMT3. Through the comprehensive analysis (membership function value), it was found that the CEI of the peat treatments (PT1–PT4) was higher than that of the rabbit manure treatments (RMT1–RMT4). Among them, PT3 had the highest CEI (0.77), and the CEI of RMT3 > 0.5 (higher than the average of all treatments, and was highest in rabbit manure treatment). Therefore, PT3 and RMT3 were selected for subsequent optimization experiment with 50% peat/rabbit manure compost + 25% perlite + 25% vermiculite (v/v/v).

**Table 4:** Characteristics of pre-proportioning growing media.

	BD (g/cm^3^)	TP (%)	AP (%)	WHP (%)	pH value	EC (mS/cm)	CEI
Peat treatment							
PT1	0.11 ± 0.01 h	68.7 ± 0.6 a	20.8 ± 2.3 ab	47.9 ± 2.8 abcd	5.96 ± 0.08 c	0.23 ± 0.01 e	0.66
PT2	0.19 ± 0.02 cd	63.1 ± 0.5 b	17.2 ± 3.1 bc	45.9 ± 3.5 bcd	5.86 ± 0.05 c	0.20 ± 0.01 fg	0.58
PT3	0.20 ± 0.00 cd	67.7 ± 0.2 a	14.0 ± 2.3 c	53.8 ± 2.5 ab	5.70 ± 0.04 d	0.18 ± 0.00 g	0.77
PT4	0.16 ± 0.02 ef	67.0 ± 3.7 a	11.9 ± 4.1 c	55.1 ± 7.8 a	5.75 ± 0.04 d	0.22 ± 0.01 ef	0.69
Rabbit manure treatment							
RMT1	0.15 ± 0.00 fg	68.8 ± 1.8 a	25.4 ± 2.4 a	43.4 ± 0.6 cd	8.21 ± 0.04 a	2.58 ± 0.02 a	0.40
RMT2	0.21 ± 0.03 bc	62.2 ± 2.5 b	20.1 ± 4.7 b	42.1 ± 7.2 d	7.83 ± 0.06 b	2.43 ± 0.01 b	0.27
RMT3	0.22 ± 0.01 bc	68.1 ± 3.2 a	17.1 ± 0.5 bc	51.0 ± 3.7 abc	7.79 ± 0.08 b	2.02 ± 0.00 d	0.54
RMT4	0.23 ± 0.00 ab	67.0 ± 0.3 a	13.3 ± 1.2 c	53.6 ± 1.5 ab	8.15 ± 0.09 a	2.30 ± 0.02 c	0.48

The characteristics include bulk density (BD), total porosity (TP), aeration porosity (AP), water holding porosity (WHP), pH value, electrical conductivity (EC), and comprehensive evaluation index (CEI). PT1–PT4 were peat treatments in which perlite, vermiculite, and peat were mixed with a ratio of 50:0:50, 33.3:33.3:33.3, 25:25:50, and 20:20:60, respectively; RMT1–RMT4 were rabbit manure treatments in which perlite, vermiculite, and rabbit manure compost were mixed with a ratio of 50:0:50, 33.3:33.3:33.3, 25:25:50, and 20:20:60, respectively. The means with different letters in a column differ significantly (p < 0.05, mean ± standard deviation, and n = 3).

### 3.2 Growing media optimization of plant bioassays

#### 3.2.1 Physicochemical properties of growing media

Physiochemical properties of different growing media with rabbit manure compost proportioning of 0%–50% are shown in **Table 5**. The BD was 0.22–0.24 g/cm^3^, which was not affected by rabbit manure compost content. The TP of growing media was all > 60%, which met the requirement of growing media for vegetables (NY/T 2118–2012). At the same time, the AP increased with rabbit manure compost. Particularly, the AP of T50 increased by 61.2% compared with CK. In contrast, WHP decreased with the increase in rabbit manure compost proportion. Still, the minimum value of 49% was also higher than the required (NY/T 2118–2012). The OM content of growing media significantly increased by 8.3%–39.9% with the addition of rabbit manure compost. However, when peat was completely replaced by rabbit manure compost, the pH value of rabbit manure compound growing media reached 7.67, and the EC value reached 2.3 mS/cm, which may have high salt stress. Nonetheless, from the perspective of nutrient supply, higher pH and EC usually mean higher nutrient input. In terms of nutrient elements, total N, P, K, Ca, and Mg contents in T10–T50 were increased by 89.3%–332.1%, 114.3%–342.9%, 14.2%–30.0%, 14.3%–122.9%, and 29.3%–178.0% compared with CK.

**Table 5:** Physical and chemical properties of different treatments.

	CK	T10	T20	T30	T40	T50
BD (g/cm^3^)	0.23 ± 0.00 a	0.23 ± 0.01 a	0.24 ± 0.02 a	0.24 ± 0.02 a	0.22 ± 0.01 a	0.23 ± 0.01 a
TP (%)	69.9 ± 2.1 a	66.5 ± 0.6 ab	68.9 ± 1.7 a	66.0 ± 3.0 ab	63.8 ± 3.0 b	70.3 ± 1.0 a
AP (%)	12.1 ± 0.0 c	12.7 ± 1.0 bc	18.8 ± 0.6 a	13.8 ± 1.5 bc	15.3 ± 1.0 b	19.5 ± 1.5 a
WHP (%)	58.0 ± 2.0 a	54.0 ± 1.0 b	51.0 ± 0.0 bcd	53.3 ± 0.6 bc	49.0 ± 3.0 d	50.7 ± 1.2 cd
OM (%)	37.3 ± 0.5 d	40.4 ± 1.2 c	47.1 ± 0.9 b	46.8 ± 1.6 b	45.7 ± 0.5 b	50.3 ± 0.9 a
pH value	5.60 ± 0.06 f	6.66 ± 0.05 e	7.05 ± 0.03 d	7.23 ± 0.05 c	7.40 ± 0.02 b	7.67 ± 0.05 a
EC (mS/cm)	0.21 ± 0.00 f	0.90 ± 0.00 e	1.36 ± 0.00 d	1.76 ± 0.00 c	1.96 ± 0.00 b	2.30 ± 0.01 a
N (%DM)	0.28 ± 0.02 f	0.53 ± 0.04 e	1.09 ± 0.01 b	0.74 ± 0.05 d	0.98 ± 0.05 c	1.21 ± 0.03 a
K (%DM)	1.40 ± 0.02 b	1.60 ± 0.18 ab	1.68 ± 0.05 a	1.63 ± 0.07 ab	1.82 ± 0.10 a	1.61 ± 0.06 ab
P (%DM)	0.07 ± 0.00 c	0.15 ± 0.00 bc	0.23 ± 0.05 ab	0.27 ± 0.05 ab	0.31 ± 0.01 a	0.28 ± 0.09 a
Ca (%DM)	0.35 ± 0.13 c	0.40 ± 0.19 bc	0.58 ± 0.02 abc	0.60 ± 0.12 abc	0.78 ± 0.17 a	0.72 ± 0.10 ab
Mg (%DM)	0.41 ± 0.22 b	0.53 ± 0.22 ab	0.85 ± 0.30 ab	0.92 ± 0.36 ab	1.14 ± 0.15 a	0.96 ± 0.29 ab

The properties include bulk density (BD), total porosity (TP), aeration porosity (AP), water holding porosity (WHP), organic matter (OM), pH, electrical conductivity (EC), nitrogen (N), phosphorus (P), potassium (K), calcium (Ca), and magnesium (Mg) contents based on dry matter (DM). CK was treatment in which rabbit manure compost, peat, perlite, and vermiculite were mixed with a ratio of 0:50:25:25; T10–T50 were treatments in which rabbit manure compost, peat, perlite, and vermiculite were mixed with a ratio of 10:40:25:25, 20:30:25:25, 30:20:25:25, 40:10:25:25, and 50:0:25:25. The means with different letters in a row differ significantly (p < 0.05, mean ± standard deviation, and n = 3).

#### 3.2.2 Effects of growing media on seed emergence, plant growth parameters, biomass, and nutrient effects

**Figure 1:**
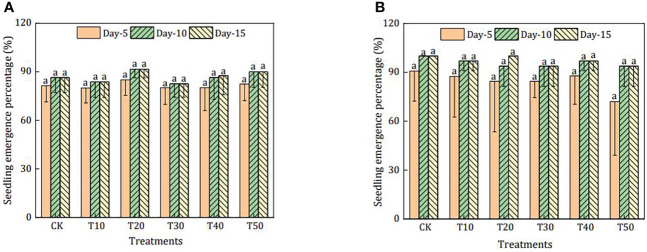
Seedling emergence percentage of different treatments for calendula **(A)** and cucumber **(B)**. CK was treatment in which rabbit manure compost, peat, perlite, and vermiculite were mixed with a ratio of 0:50:25:25; T10–T50 were treatments in which rabbit manure compost, peat, perlite, and vermiculite were mixed with a ratio of 10:40:25:25, 20:30:25:25, 30:20:25:25, 40:10:25:25, and 50:0:25:25. The means with different superscript letters for a bar differ significantly (*p* < 0.05, *n* = 80 for calendula and *n* = 70 for cucumber), and error bars represent standard deviation.

The emergence of seedlings in the growing media during the different periods is shown in **Figure 1**. For calendula, the seedling emergence percentage was not significantly affected by the rabbit manure compost proportion and cultivation period, and the highest emergence (91.6%) was observed in T20. Similarly, the seedling emergence percentage of cucumber was comparable in different media and periods, ranging from 93.8% to 100%. However, the emergence of cucumber was delayed by rabbit manure compost on the 5th day.

**Figure 2:**
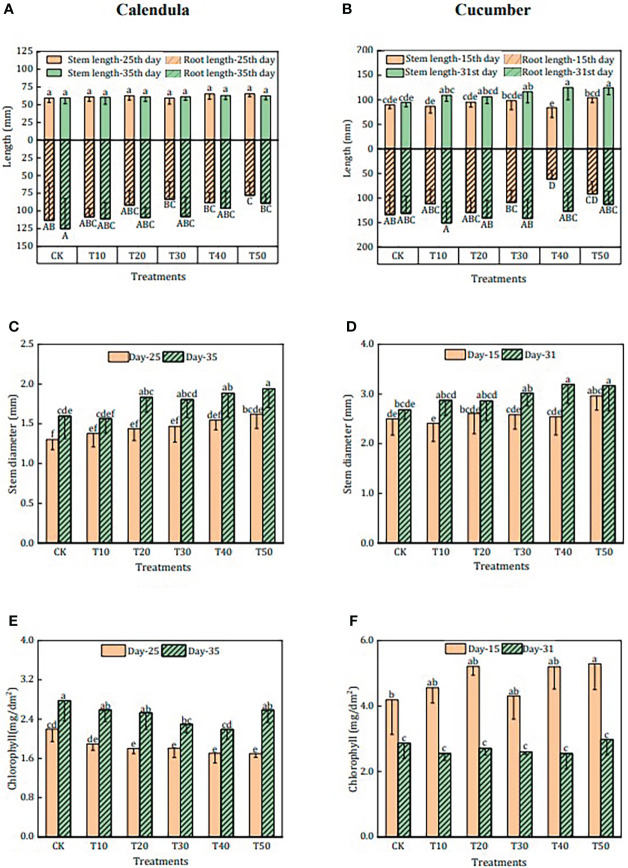
Effects of different treatments on the plant growth parameters of calendula and cucumber. CK was treatment in which rabbit manure compost, peat, perlite, and vermiculite were mixed with a ratio of 0:50:25:25; T10–T50 were treatments in which rabbit manure compost, peat, perlite, and vermiculite were mixed with ratio of 10:40:25:25, 20:30:25:25, 30:20:25:25, 40:10:25:25, and 50:0:25:25. The length of stem and root of calendula and cucumber are shown in **(A)** and **(B)** Stem diameter of calendula and cucumber is shown in **(C)** and **(D)** Chlorophyll of calendula and cucumber is shown in **(E)** and **(F)** The means with different superscript letters for a bar differ significantly (*p* < 0.05, *n* = 10 for calendula and *n* = 8 for cucumber), and error bars represent standard deviation.

The plant growth parameters of the different growing media are shown in **Figure 2**. For calendula, there was no significant difference in stem length and root length among the growing media with different rabbit manure compost contents (**Figure 2A**). Only the stem diameter (**Figure 2C**) was increased up to 21.3% (in T50) with rabbit manure compost compared to CK. Regarding the cultivation period, the stem diameter of T10–T50 was increased by 13.8%–27.1% from the 25th day to the 35th day. Furthermore, the chlorophyll content (**Figure 2E**) of calendula increased by 26.4%–52.2% during the cultivation period.

In contrast to calendula, the stem length (**Figure 2B**) of cucumber in T30, T40, and T50 was significantly higher than those of CK. On the 31st day, the stem diameter (**Figure 2D**) in T40 and T50 was 18.3% and 19.0% higher than that in CK. The total chlorophyll content of cucumber leaves decreased with the cultivation period (**Figure 2F**). Finally, total chlorophyll content on the 31st day was not significantly different among all growing media.

**Figure 3:**
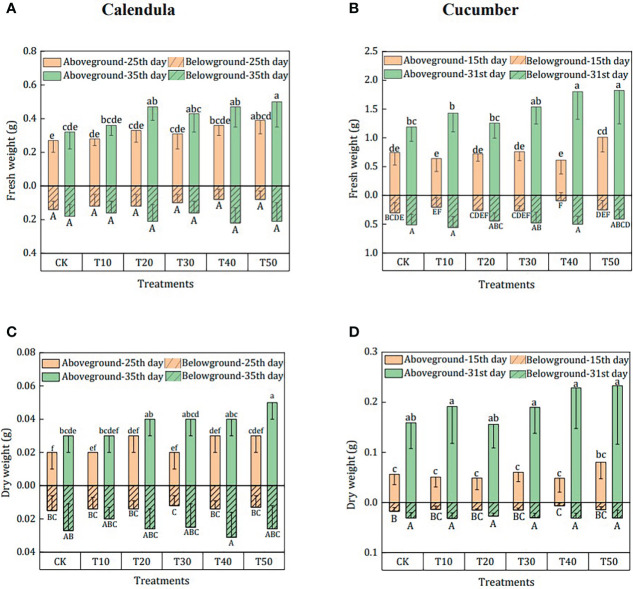
Effects of different treatments on the plant biomass of calendula and cucumber. CK was treatment in which rabbit manure compost, peat, perlite, and vermiculite were mixed with a ratio of 0:50:25:25; T10–T50 were treatments in which rabbit manure compost, peat, perlite, and vermiculite were mixed with a ratio of 10:40:25:25, 20:30:25:25, 30:20:25:25, 40:10:25:25, and 50:0:25:25. Aboveground and belowground fresh weight of calendula and cucumber are shown in **(A)** and **(B)** Aboveground and belowground dry weight of calendula and cucumber are shown in **(C)** and **(D)** The means with different superscript letters for a bar differ significantly (*p* < 0.05, *n* = 10 for calendula and *n* = 8 for cucumber), and error bars represent standard deviation.

The plant biomass in different growing media is shown in **Figure 3**. For calendula, there was no significant difference in fresh belowground weight (**Figure 3A**) and dry belowground weight (**Figure 3C**) in different growing media and cultivation period. The only exception was that the belowground dry weight of T40 increased by 121.4% from the 25th to the 35th day. At the same time, there was some noticeable difference in aboveground biomass, especially the fresh aboveground weight (**Figure 3A**) of T40 and T50, which were about 50% higher than that in CK. Similarly, fresh aboveground weight (**Figure 3B**) of cucumber on the 31st day of T40 and T50 was 51.3% and 52.9% higher than the CK. The corresponding dry aboveground weight (**Figure 3D**) of T40 and T50 both increased by 43.8%, but there was no significant difference in the belowground biomass between these growing media. Regarding the cultivation period, both the aboveground and belowground biomass significantly increased from the 15th day to the 31st day. For example, the aboveground and belowground fresh weights in T40 increased by 1.95 times and 4.56 times, respectively.

The nutrient performance of plants aboveground in different growing media is shown in **Table 6**. For calendula, there was no significant difference in K content with different growing media, but the contents of N, P, and Mg in T10–T50 increased by 6.0%–33.6%, 31%–141.4%, and 80.4%–107.8% compared with CK. The contents of Ca in T10 and T20 were 50.4% and 37.7% higher than in CK, but those in T40 and T50 were 12.3%–30.2% lower than those in CK. There was no significant difference in N, K, and Ca content for cucumbers with different growing media, but the contents of P and Mg in T10–T50 increased by 82.6%–117.4% and 35.1%–67.6%. Overall, using rabbit manure compost could promote the absorption of nutrients by plants, especially P and Mg, which may be responsible for increased plant biomass.

**Table 6:** Effects of different treatments on the aboveground macronutrient of calendula and cucumber (35th day for calendula and 31st day for cucumber).

	Calendula					Cucumber				
	N	P	K	Ca	Mg	N	P	K	Ca	Mg
	% Dry matter									
CK	1.34 c	0.29 c	4.23 a	2.68 b	0.51 b	1.23 a	0.23 b	2.21 a	1.37 ab	0.37 b
T10	1.42 c	0.70 a	4.10 a	4.03 a	0.92 a	1.25 a	0.50 a	2.56 a	1.52 a	0.50 ab
T20	1.56 b	0.51 ab	4.26 a	3.69 a	1.06 a	1.15 a	0.50 a	2.88 a	1.37 ab	0.53 ab
T30	1.79 a	0.44 bc	4.84 a	2.65 b	0.97 a	1.17 a	0.46 ab	3.23 a	1.24 ab	0.55 ab
T40	1.58 b	0.40 bc	4.80 a	2.35 c	1.06 a	1.14 a	0.42 ab	3.52 a	1.06 ab	0.56 ab
T50	1.74 a	0.38 bc	5.38 a	1.87 c	1.05 a	1.32 a	0.50 a	4.02 a	0.93 b	0.62 a

CK was treatment in which rabbit manure compost, peat, perlite, and vermiculite were mixed with a ratio of 0:50:25:25; T10–T50 were treatments in which rabbit manure compost, peat, perlite, and vermiculite were mixed with a ratio of 10:40:25:25, 20:30:25:25, 30:20:25:25, 40:10:25:25, and 50:0:25:25. The means (n = 3) with different letters in a column differ significantly (p < 0.05).

#### 3.2.3 Comprehensive evaluation of growing media

The stem/root length, stem diameter, chlorophyll, aboveground fresh/dry weight, and macronutrient (e.g., N, P, Ca, and Mg) of calendula and cucumber significantly changed with the addition of rabbit manure compost. These indicators could reflect most of the growth information on seedlings. Therefore, they were selected for the final minimum data set as the comprehensive evaluation indicators of growing media quality, and the calculation results are shown in **Table 7**. The CEI of rabbit manure compound growing media was all higher than CK in the cultivation of calendula and cucumber. For calendula, the CEI of T20–T50 was > 0.5 (above the average). This indicates that the growth of calendula was significantly promoted when the proportion of rabbit manure compost was ≥ 20%. For cucumber, the CEI of T10, T30, T40, and T50 were all > 0.5 (above the average). Particularly, prominent advantages were found in stem length, stem diameter, aboveground fresh/dry weight, and N and Mg content when rabbit manure compost was added. The best performance was found at T50 for both plants, and the proportion of 30%–50% (that is, 60%–100% instead of peat) was suitable according to the comprehensive evaluation.

**Table 7:** A comprehensive evaluation of calendula and cucumber seedling cultivation with different treatments.

	Stem length	Root length	Stem diameter	Chlorophyll	Aboveground dry weight	Aboveground fresh weight	N	P	Ca	Mg	CEI
Calendula											
CK	0.00	1.00	0.08	1.00	0.00	0.00	0.00	0.00	0.38	0.00	0.25
T10	0.22	0.61	0.00	0.67	0.00	0.22	0.18	1.00	1.00	0.75	0.47
T20	0.48	0.56	0.71	0.58	0.50	0.83	0.49	0.54	0.84	1.00	0.65
T30	0.50	0.53	0.64	0.17	0.50	0.61	1.00	0.37	0.36	0.84	0.55
T40	1.00	0.20	0.85	0.00	0.50	0.83	0.53	0.27	0.22	1.00	0.54
T50	0.84	0.00	1.00	0.67	1.00	1.00	0.89	0.22	0.00	0.98	0.66
Cucumber											
CK	0.00	0.48	0.00	0.74	0.04	0.00	0.50	0.00	0.75	0.00	0.25
T10	0.47	1.00	0.37	0.00	0.46	0.38	0.61	0.49	1.00	0.52	0.53
T20	0.38	0.72	0.35	0.37	0.00	0.11	0.06	0.85	0.75	0.64	0.42
T30	0.71	0.76	0.66	0.12	0.44	0.55	0.17	1.00	0.53	0.72	0.56
T40	1.00	0.36	1.00	0.00	0.94	0.97	0.00	0.09	0.22	0.76	0.53
T50	0.99	0.00	0.95	1.00	1.00	1.00	1.00	0.48	0.00	1.00	0.74

N, nitrogen; P, phosphorus; Ca, calcium; Mg, magnesium. CK was treatment in which rabbit manure compost, peat, perlite, and vermiculite were mixed with a ratio of 0:50:25:25; T10–T50 were treatments in which rabbit manure compost, peat, perlite, and vermiculite were mixed with a ratio of 10:40:25:25, 20:30:25:25, 30:20:25:25, 40:10:25:25, and 50:0:25:25. The value in the first 10 columns is the membership function value of the specific plant parameter in the specific treatment. The comprehensive evaluation index (CEI) is the mean of the membership function values of 10 plant parameters. Dark gray shading: membership function value = 0.7–1.0; light gray shading: membership function value = 0.5–0.7; no shading: membership function value = 0–0.5.

### 3.3 Greenhouse gas emission reduction

The greenhouse gas emission from peat extraction would be reduced by manufacturing rabbit manure compost as a growing medium for transplanting crops. The following is a preliminary calculation: in China, the annual output of rabbit manure was 9.28 × 10^6^–2.29 × 10^7^ t (Peng and Wang, 2004; Nation Bureau of Statistics, 2021). This could produce 7.44 × 10^6^–3.55 × 10^7^ m^3^ of rabbit manure compost [considering the measured bulk density of fresh rabbit manure was 525 kg/m^3^, and the compost loss was 18.5%–57.9% of the initial volume (Breitenbeck and Schellinger, 2004)]. Based on the results of this study that peat could be replaced by 60%–80% rabbit manure compost (T30–T50), then the total amount of peat that could be replaced was 4.46 × 10^6^–3.55 × 10^7^ m^3^/year. The greenhouse gas emission coefficient of peat extraction is 81.76–11,437.77 g CO_2_-equivalent/m^3^ [the peat extract depth is 0.2–10.2 m (Tiemeyer et al., 2016), and the greenhouse gas emission coefficient of peat extraction is 408.79–1,121.35 g CO_2_-equivalent/m^2^ (Maljanen et al., 2010)]. Replacing peat with rabbit manure compost would reduce greenhouse gas emissions by 3.65 × 10^5^–4.06 × 10^8^ kg CO_2_-equivalent/year.

## 4 Discussion

### 4.1 Optimized proportion of rabbit manure compound growing media

The results of pre-optimization of the physicochemical properties of the growing media showed that the performance of the growing media could be influenced by the mixing ratios whether it was the rabbit manure treatment or the peat treatment (**Table 4**). It indicated the importance of mixing ratio for the production of the growing media, while the CEIs of the four rabbit manure treatments (RMT1–RMT4) where peat was completely replaced were lower than those of the peat treatments (PT1–PT4). The main reason could be attributed to the higher pH and EC values in rabbit manure treatments, which is often a concern when using manure as a substrate. However, the higher BD and AP of the rabbit manure treatments indicate that the growing media has better stability and ventilation performance. After comprehensive evaluation, 25% perlite and 25% vermiculite are the combination of excipients with suitable physical and chemical properties. After the excipients were determined, peat was replaced by rabbit manure compost to seek an optimized proportion of rabbit manure compound growing media. It was shown that rabbit manure compound growing media had similar or better properties (e.g., porosity, bulk density, OM, and nutrition) compared to CK, which represented standard peat media (**Table 5**). The exception was the high EC value. This result is consistent with previous studies (Chang et al., 2021; Ikrarwati et al., 2021).

Appropriate physicochemical properties are required, but the most critical in the optimization of the ratio of the rabbit manure compound growing media would be the seedling effects, which is a direct and objective evaluation. The final seedling effects of rabbit manure compound growing media on salinity-tolerant calendula and salt-intolerant cucumber indicated that the emergence of seedlings was not affected by high-salinity rabbit manure compost, and even the growth of the seedlings was promoted. In particular, the appropriate proportion of rabbit manure compost was 30%–50% (that is, 60%–100% instead of peat). Similarly, the best seedling performance of cabbage (*Brassica rapa* var. *glabra* Regel) was achieved when the rabbit manure compost ratio was 30% (Li et al., 2022). It can be seen that rabbit manure compost can be used as a substitute for peat, regardless of crop type. Additionally, Adekiya et al. (2020) and Pereira et al. (2020) also reported positive plant responses in the nursery under the application of rabbit manure. This may be due to the good physical properties and fertilizer effects of the rabbit manure growing media (Cabanillas et al., 2013).

Using rabbit manure compost instead of 60%–100% peat to prepare the growing media could reduce greenhouse gas emissions by 3.65 × 10^5^–4.06 × 10^8^ kg CO_2_-equivalent/year in China. Moreover, carbon sequestration would be effectively increased, and carbon dioxide emissions would also be reduced by transplanting seedlings containing rabbit manure compound growing media to soil (Ondoño et al., 2015; Banik et al., 2021). Such huge greenhouse gas emission reduction potential encourages industrial development and technological progress. It would be valuable to use waste as a substitute for peat. For example, in this study, we attempted to use rabbit manure compound growing media to cultivate calendula and cucumber.

### 4.2 Correlation between growing media characteristics and plant growth performance

**Figure 4:**
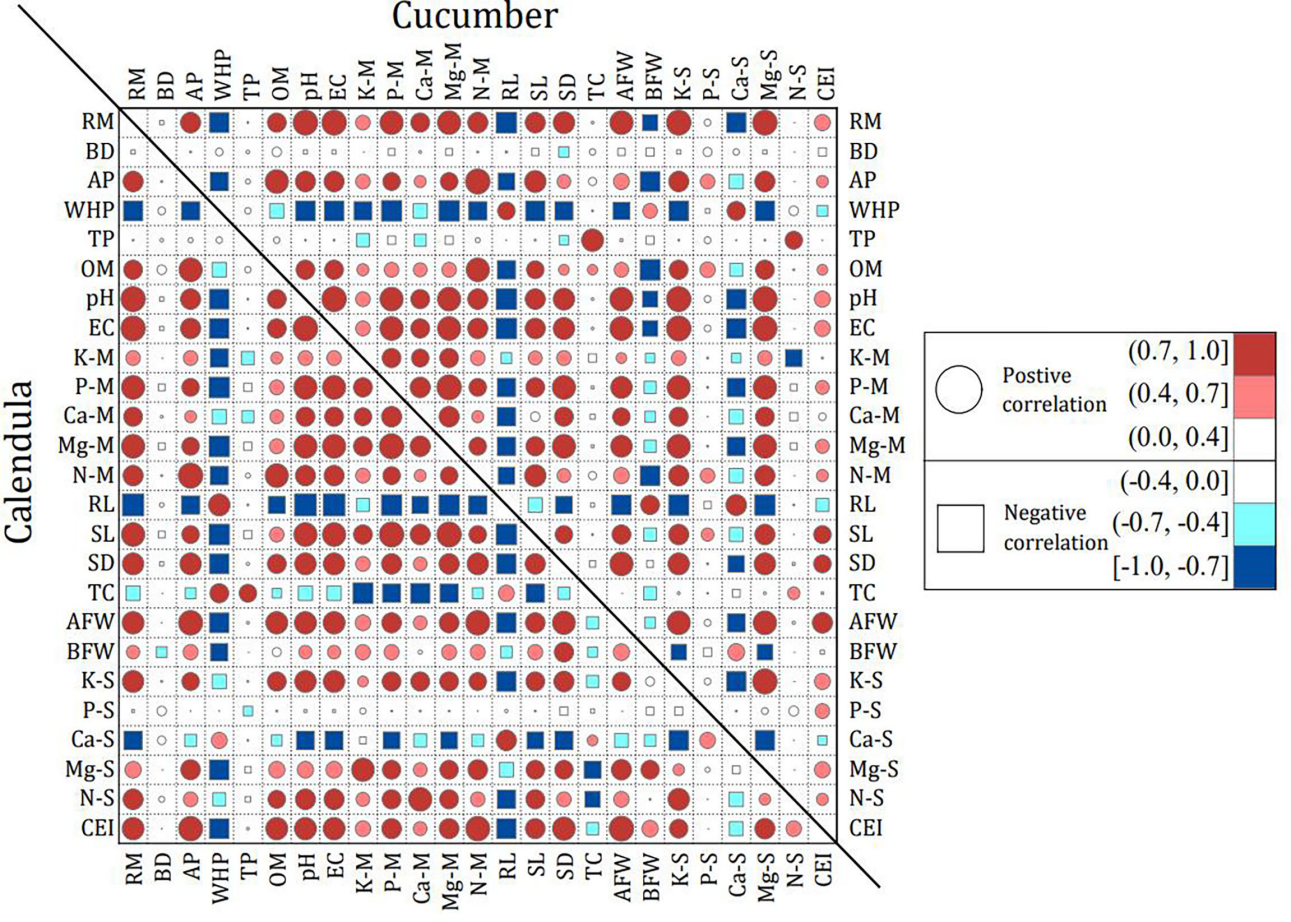
Correlation between rabbit manure compound growing media properties and plant growth performance of calendula and cucumber. The properties of rabbit manure compound growing media include rabbit manure content (RM), bulk density (BD), aeration porosity (AP), water holding porosity (WHP), total porosity (TP), organic matter (OM), pH, electrical conductivity (EC), and nutrient elements of the media (including N-M, P-M, K-M, Ca-M, and Mg-M; refer to total nitrogen, phosphorus, potassium, calcium, and magnesium of the media). The plant growth performance includes root length (RL), stem length (SL), stem diameter (SD), total chlorophyll (TC), aboveground fresh weight (AFW), belowground fresh weight (BFW), the macronutrient of seedling (including N-S, P-S, K-S, Ca-S, and Mg-S; refer to total nitrogen, phosphorus, potassium, calcium, and magnesium of the aboveground seedling), and the comprehensive evaluation index (CEI) of seedlings. Positive and negative correlation coefficients are presented as circles and squares, respectively. Their magnitude ranges are indicated by the colors shown in the legend, with increments within each magnitude range indicated by the size of circles and squares.

Spearman’s correlation coefficients between rabbit manure compound growing media characteristics and plant growth performance (on the 35th day for calendula and the 31st day for cucumber) were calculated and shown in **Figure 4**. For the physicochemical characteristics of growing media, the proportion of rabbit manure compost was positively correlated with AP, OM, and nutrient element content of the growing media, indicating that the properties of the growing media could be improved by rabbit manure compost. This may be because of the high lignocellulose and nutrient content of rabbit manure compost (Tognetti et al., 2007).

For calendula, the AP and OM of the media were positively correlated with the CEI. In other words, the improved seedling effects (e.g., stem diameter and aboveground biomass) were attributed to the increasing AP and OM contents upon adding rabbit manure compost. The EC value was negatively correlated with the root length with Spearman’s correlation coefficient of −1.00. This means higher EC values were not conducive to the growth of roots, which is consistent with the report of Salomon et al. (2021) because plants under high salinity would be subjected to osmotic stress. This could hinder the water absorption of seedling roots. However, there was a strong positive correlation between the EC value and the CEI, which may be that a high EC represents more nutrient content. At the same time, some studies have reported that the EC value is less critical for plant growth than physical properties (Chong et al., 1994). Furthermore, leaching may easily occur in rabbit manure compound growing media with high porosity (Zhang et al., 2012). Additionally, nutrient elements (e.g., K, P, Ca, Mg, and N) in the growing rabbit manure compound growing media strongly correlated with the CEI. There was a positive effect on stem length (Spearman’s correlation coefficients were 0.77–1.00), stem diameter (Spearman’s correlation coefficients were 0.54–0.89), and aboveground biomass (Spearman’s correlation coefficients were 0.55–0.99). According to Berti et al. (2017), the K element can participate in enzyme activation and protein synthesis under stress conditions (for example, salt stress), thereby enhancing plant stress resistance. Similarly, Mg and N engage in many processes in plant photosynthesis, respiration, carbohydrate, and protein synthesis (Silva et al., 2017; Pereira et al., 2020). At the same time, P provides energy for plant development (Ikrarwati et al., 2021), showing that the growth performance of seedlings might be due to the increase of nutrients in the rabbit manure compound growing media. This result indicated that seedling effects could be promoted by enhancing the nutrition of growing media. Of course, there was also the possibility of nutritional deficiency in CK. From this perspective, an additional advantage of rabbit manure compost as a growing media was that it had potential to lower fertilizer requirements.

For cucumber, the correlations of growing media properties and seedling growth parameters were similar to those of calendula. There was a positive correlation between rabbit manure compost content and CEI of cucumber seedlings, especially in stem length, stem diameter, and the contents of K and Mg (Spearman’s correlation coefficients were 0.83–1.00). Therefore, the increase in rabbit manure compost was beneficial to the growth of the aboveground cucumber seedlings. Similarly, the positive impact of rabbit manure on tomato stem length, stem diameter, and leaf number has already been shown in studies by Mahmoud et al. (2014).

The results of the correlation analysis showed that good seedling effects could be obtained using biomass growing media. The aboveground biomass of seedlings could be improved, and nutrients could be input by increasing the OM content, aeration, porosity, and nutrient elements.

## 5 Conclusion and further directions

Our results showed that adding rabbit manure compound was an effective method of replacing peat to manufacture growing media during the cultivation of calendula and cucumber, and the proportion of 30%–50% (that is, 60%–100% instead of peat) was more suitable. The seedling effects were promoted due to the appropriate physical and chemical properties (e.g., OM content, aeration porosity, and nutrient elements) of the rabbit manure compound growing media. Moreover, the greenhouse gas emissions from peat extraction would be reduced by manufacturing waste as a growing medium for transplanting crops. The greenhouse gas emission reduction potential using rabbit manure compost replacing peat would be 3.65 × 10^5^–4.06 × 10^8^ kg CO_2_-equivalent/year in China. Further research is required, including related fertilizer management for different crops, to achieve a win–win situation between rabbit manure resource utilization and carbon emission reduction.

## Data availability statement

The original contributions presented in the study are included in the article/supplementary material. Further inquiries can be directed to the corresponding author.

## Author contributions

Conceptualization, RL and HW; methodology, LW; software, HH and CY; writing—original draft preparation, RL; writing—review and editing, HW and LW; supervision, HW; funding acquisition, HW. All authors have read and agreed to the published version of the manuscript.

## Funding

This study received funding from the China Agriculture Research System of MOF and MARA (CARS - 43 - D - 3).

## Conflict of interest

The authors declare that the research was conducted in the absence of any commercial or financial relationships that could be construed as a potential conflict of interest.

## Publisher’s note

All claims expressed in this article are solely those of the authors and do not necessarily represent those of their affiliated organizations, or those of the publisher, the editors and the reviewers. Any product that may be evaluated in this article, or claim that may be made by its manufacturer, is not guaranteed or endorsed by the publisher.

## Abbreviations

EC, electrical conductivity; BD, bulk density; TP, total porosity; AP, aeration porosity; WHP, water holding porosity; CEI, comprehensive evaluation index; OM, organic matter; DM, dry matter; N, nitrogen; P, phosphorus; K, potassium; Ca, calcium; Mg, magnesium; PT1–PT4 were peat treatments in which perlite, vermiculite, and peat were mixed with a ratio of 50:0:50, 33.3:33.3:33.3, 25:25:50, and 20:20:60, respectively; RMT1–RMT4 were rabbit manure treatments in which perlite, vermiculite, and rabbit manure compost were mixed with a ratio of 50:0:50, 33.3:33.3:33.3, 25:25:50, and 20:20:60, respectively; CK was treatment in which rabbit manure compost, peat, perlite, and vermiculite were mixed with a ratio of 0:50:25:25; T10–T50 were treatments in which rabbit manure compost, peat, perlite, and vermiculite were mixed with a ratio of 10:40:25:25, 20:30:25:25, 30:20:25:25, 40:10:25:25, and 50:0:25:25.
